# Supplementary material for: Enhancing Existing Formal Home Care to Improve and Maintain Functional Status in Older Adults: Results of a Feasibility Study on the Implementation of Care to Move (CTM) in an Irish Healthcare Setting
Source: Int J Environ Res Public Health. 2022 Sep 6;19(18):11148. doi: 10.3390/ijerph191811148 (PMC9517683; doi:10.3390/ijerph191811148)

Supplementary Material
Horgan et al.

Table S1: Secondary Outcome Scores T1-T3 – Rockwood Frailty Score, Phone-FITT, SF-36 Quality of Life Scores

	Time 1 Baseline	Time 2 at 8 weeks	Time 3 at X-X months
Rockwood Frailty Score $: n	35	21	13
Mean (SD)	5.4 (0.8)	5.6 (0.6)	5.5 (0.7)
Median (IQR)	*6.0 (1.0)*	*6.0 (1.0)*	*6.0 (1.0)*
Cohens d_s	_	0.04	0.03
Phone-FITT Household FD (score) ¶: n	34	21	13
Mean (SD)	11.1 (9.9)	9.0 (8.2)	9.7 (9.5)
Median (IQR)	*8.5 (18.0)*	*10.0 (12.0)*	*6.0 (18.0)*
Cohens d_s	_	-0.23[a]	-0.14
Phone-FITT Recreational FD (score) ¶: n	34	21	13
Mean (SD)	10.2 (8.9)	12.9 (8.8)	14.7 (6.8)
Median (IQR)	*8.0 (8.5)*	*11.0 (14.0)*	*13.0 (11.0)*
Cohens d_s	_	0.30[a]	**0.53[b]**
Phone-FITT Total FD (Score) ¶: n	34	21	13
Mean (SD)	21.8 (18.5)	21.9 (13.2)	24.4 (11.7)
Median (IQR)	*16.0 (19.5)*	*20.0 (18.0)*	*22.0 (16.0)*
Cohens d_s	_	0.00	0.15
Phone-FITT Household FDI: n	34	21	13
Mean (SD)	13.1 (11.2)	10.7 (9.4)	11.2 (10.6)
Median (IQR)	*11.0 (20.0)*	*13.0 (15.0)*	*8.0 (20.0)*
Cohens d_s	_	-0.23[a]	-0.17
Phone-FITT Recreational FDI: n	34	21	13
Mean (SD)	11.4 (9.9)	14.6 (9.8)	16.3 (7.6)
Median (IQR)	*8.0 (11.0)*	*13.0 (15.0)*	*15.0 (14.0)*
Cohens d_s	_	**0.32[a]**	**0.53[b]**
Phone-FITT Total FDI:	34	21	13
Mean (SD)	24.5 (17.6)	25.2 (14.7)	27.5 (12.8)
Median (IQR)	18.5 (25.3)	26.0 (18.0)	24.0 (17.0)
Cohens d_s	_	-0.04	-0.18
SF-36 Physical functioning:	34	19	13
**Mean** (SD)	14.1 (7.5)	12.4 (7.5)	11.5 (9.4)
Median (IQR)	10.0 (75.0)	10.0 (10.0)	10.0 (10.0)
Cohens d_s	-	-0.15	-0.21
SF-36 Role limitations due to physical health:			
**Mean** (SD)	35	19	13
Median (IQR)	30.7 (42.1)	44.7 (42.1)	53.8 (46.6)
Cohens d_s	25.0 (75.0)	25.0 (100.0)	50.0 (100.0)
	-	0.36	0.57
SF-36 Role limitations due to emotional			
problems:	35	19	13
**Mean** (SD)	62.9 (30.2)	84.2 (30.2)	74.6 (36.6)
Median (IQR)	100.0 (66.7)	100.0 (33.3)	100.0 (66.7)
Cohens d_s	-	0.55	0.28
SF-36 Energy / fatigue:	35	19	13
**Mean** (SD)	41.6 (23.4)	49.5 (23.4)	44.6 (20.4)

Median (IQR)	35.0 (30.0)	50.0 (25.0)	40.0 (35.0)
Cohens d_s	-	0.33	0.13
SF-36 Emotional well-being:	35	19	13
**Mean** (SD)	64.3 (18.2)	73.9 (18.2)	80.0 (14.5)
Median (IQR)	68.0 (36.0)	76.0 (28.0)	80.0 (12.0)
Cohens d_s	-	0.41	0.68
SF-36 Social functioning:	34	19	13
**Mean** (SD)	53.1 (23.3)	73.0 (25.8)	75.0 (27.5)
Median (IQR)	45.0 (55.0)	75.0 (37.5)	75.0 (37.5)
Cohens d_s	-	0.63	0.67
SF-36 Pain:	35	19	13
**Mean** (SD)	53.1 (23.3)	55.5 (22.4)	52.3 (25.5)
Median (IQR)	45.0 (55.0)	57.4 (50.0)	45.0 (27.5)
Cohens d_s	-	0.0.09	-0.03
SF-36 general health:	33	19	13
**Mean** (SD)	50.6 (22.4)	57.4 (22.4)	56.9 (28.0)
Median (IQR)	45.0 (40.0)	55.0 (40.0)	55.0 (50.0)
Cohens d_s	-	0.28	0.24

Key: SD Standard deviation; IQR Interquartile range;
Rockwood Frailty Score – lower score is better
Phone-FITT FD is Frequency, Duration; FDI Frequency, Duration, Intensity - higher score is better;
SF-36 - higher score is better;
Cohens d_s effect sizes – [a]small 0.2-0.5, [b]medium 0.5-0.8, [c]large 0.8-1.

Table S2: Barriers to Physical Activity/ Moving More T1-T3

	Afraid of fall/injury			*Lack of company*			*Poor Health*			*Lack of interest*		
	n	*Number saying partially agree or agree*	*% of sample*	*n*	*Number saying partially agree or agree*	*% of sample*	*n*	*Number saying partially agree or agree*	*% of sample*	*n*	*Number saying partially agree or agree*	*% of sample*
T1	*34*	*21*	*61.8*	*34*	*20*	*58.8*	*34*	*19*	*55.9*	*34*	*7*	*20.6*
T2 (8 weeks)	*17*	*10*	*58.8*	*17*	*10*	*58.8*	*16*	*6*	*37.5*	*16*	*0*	*0.0*
T3 (>6 months)	*10*	*6*	*60.0*	*10*	*4*	*40.0*	*10*	*6*	*60.0*	*10*	*1*	*10.0*

Table S3: Outcome Expectations T1-T3

	**Time 1 Baseline**		**Time 2 at 8 weeks**		**Time 3 at X-X months**	
	n	% choosing Agree or Strongly Agree	n	% choosing Agree or Strongly Agree	n	% choosing Agree or Strongly Agree
**Physical Outcome Expectations**						
Ability to perform ADLs	34	82.4	16	87.5	10	90.0
Overall body function	34	91.2	16	93.8	10	100.0
Strengthen my bones	34	64.7	16	87.5	10	60.0
Increase muscle strength	34	85.3	16	93.8	10	70.0
**Social Expectations**						
Help me socialise more	34	61.8	16	75.0	10	70.0
**Self-evaluation Outcome Expectations**						
Manage stress	34	55.9	16	68.8	10	80.0
Help my mood	34	70.6	16	87.5	10	80.0
Sense of personal accomplishment	34	85.3	16	93.8	10	90.0

Table S4: Intention and planning scale T1-T3

	**Time 1 Baseline**		**Time 2 at 8 weeks**		**Time 3 at X-X months**	
	n	% choosing A Little True or Absolutely True	n	% choosing A Little True or Absolutely True	n	% choosing A Little True or Absolutely True
**Intention**						
As part of my daily routine	34	91.2	16	100.0	9	88.9
Before I try walking	34	38.2	16	87.5	9	66.7
As part of my leisure time	34	82.4	16	93.8	9	77.8
**Planning**						
Precisely when to move more	34	26.5	16	75.0	9	44.4
Precisely where to move more	34	29.4	16	75.0	9	44.4
To continue moving more even when I feel limited by poor health	34	20.6	16	50.0	9	33.3

Figure S1: Barriers to Moving More over the 6-month timeframe

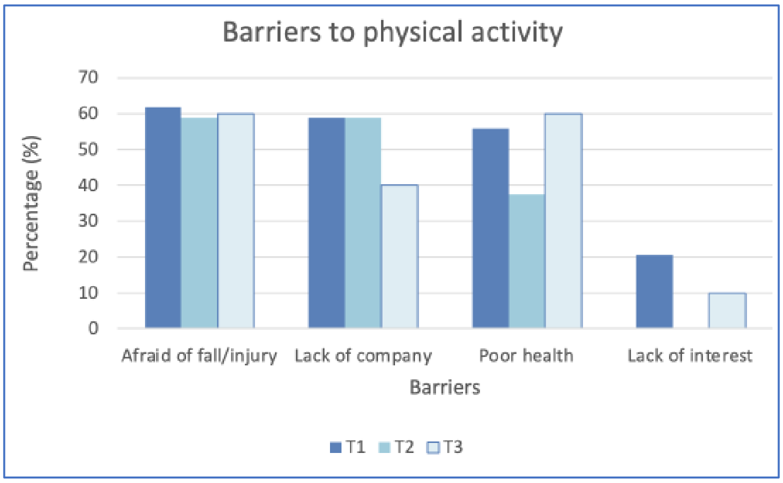

Figure S2: Outcomes expectations over the 6-month timeframe

Percentage positive responses were averaged for Q1-4 for physical outcomes expectations and for Q6-8 for self-evaluation outcome expectations for T1-T3 for figure below.

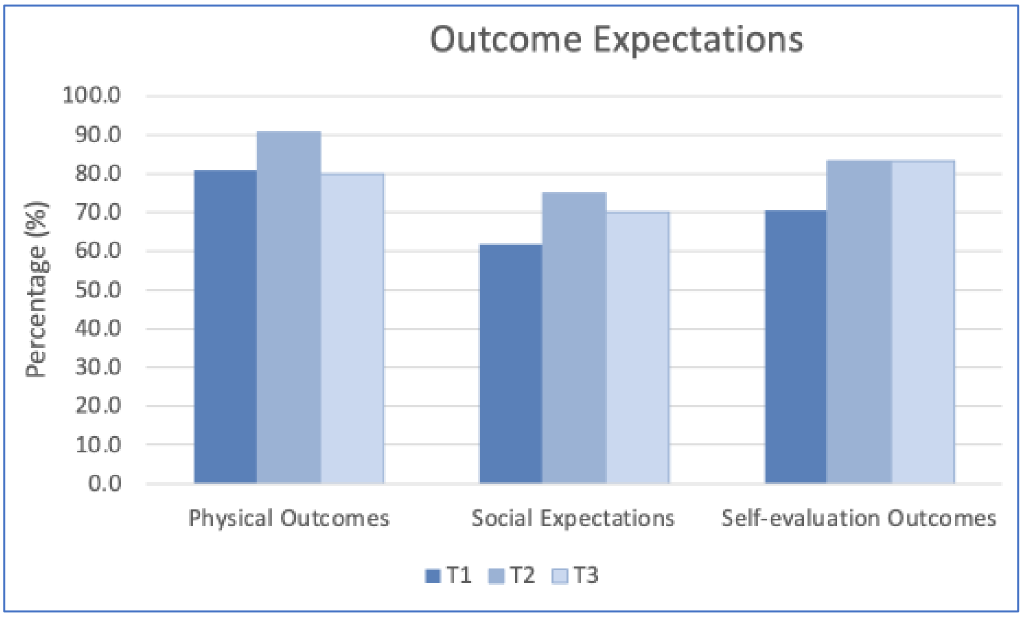

Figure S3: Intention and planning over the 6-month timeframe

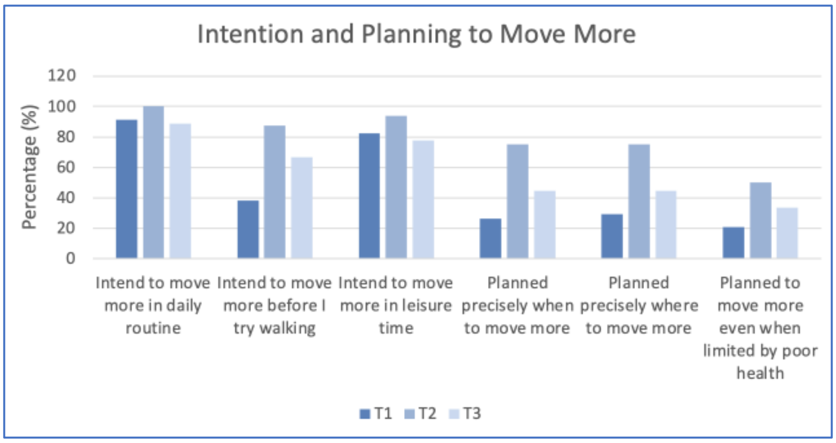

Supplement: Supplementary file 1 [file ijerph-19-11148-s001.zip › ijerph-1870959-supplementary.pdf]
